# Illawarra Born cross-generational health study: feasibility of a multi-generational birth cohort study

**DOI:** 10.1186/s40814-019-0418-5

**Published:** 2019-02-26

**Authors:** Michelle L. Townsend, Megan A. Kelly, Judy A. Pickard, Theresa A. Larkin, Victoria M. Flood, Peter Caputi, Ian M. Wright, Alison Jones, Brin F. S. Grenyer

**Affiliations:** 1 Illawarra Health and Medical Research Institute, University of Wollongong, Wollongong, NSW 2522 Australia; 2 School of Psychology, University of Wollongong, Wollongong, NSW 2522 Australia; 3 Graduate School of Medicine, University of Wollongong, Wollongong, NSW 2522 Australia; 4 Illawarra Shoalhaven Local Health District, NSW Health, Locked Mail Bag 8808, South Coast Mail Centre, North Sydney, NSW 2521 Australia; 5 Faculty of Health Sciences, University of Sydney, PO Box 170, Lidcombe, NSW 1825 Australia; 6 St Vincent’s Hospital, 390 Victoria Street, Darlinghurst, NSW 2010 Australia; 7 School of Chemistry and Molecular Biosciences, University of Wollongong, Wollongong, NSW 2522 Australia

**Keywords:** Birth cohort, Pilot study, Recruitment, Developmental origins of health and disease, Cross-generation, Mental health

## Abstract

**Background:**

There is a strong interest in the concept of developmental origins of health and disease and their influence on various factors “from cradle to grave”. Despite the increasing appreciation of this lifelong legacy across the human life course, many gaps remain in the scientific understanding of mechanisms influencing these formative phases. Cross-generational susceptibility to health problems is emerging as a focus of research in the context of birth cohort studies.

The primary aim of the Illawarra Born study is to make scientific discoveries associated with improving health and wellbeing across the lifespan, with a particular focus on preventable chronic diseases, especially mental health. This birth cohort study will follow and collect data from three cohorts representing different stages across the lifespan: infants, adults (parents) and older adults (grandparents). The multi-generational, cross-sectional and longitudinal design of this birth cohort study supports a focus on the contributions of genetics, environment and lifestyle on health and wellbeing. The feasibility of conducting a multi-generational longitudinal birth cohort project was conducted through a small pilot study.

**Methods/design:**

The purpose of this paper is to report on the feasibility and acceptability of the research protocol for a collaborative cross-generation health study in the community and test recruitment and outcome measures for the main study. This feasibility study included pregnant women who were intending to give birth in the Illawarra-Shoalhaven region in Eastern Australia. The area includes a large, regional referral hospital, with capacity to treat specialist and complex cases. Pregnant women were asked to participate in five data collection waves beginning at 22 weeks gestation and ending with a 6-month post-partum appointment. Recruitment was then extended, via the pregnant women, to also include fathers and maternal grandmothers.

**Discussion:**

This feasibility study focused on the perinatal period and collected data across three multi-disciplinary domains including mental health, diet, exposures to toxins and the role of these in maternal and infant outcomes. Forty-one families participated in extensive data collection from 22 weeks gestation to 6-months post-partum. Factors impacting on viability and feasibility including recruitment solutions provide the basis for a large-scale study.

## Background

Pregnancy is an ideal time during which to recruit women to participate in a multi-generational health and wellbeing study. Women regularly visit their physicians during pregnancy, which provides a number of opportunities to meet with and educate potential research participants. It is also a time when women may feel more motivated to contribute to research examining the influence of maternal health and wellbeing on foetal development [1]. The literature identifies this period as highly susceptible to many maternal influences [2]. Optimum nutrition, exercise and access to clean water and environments during pregnancy can result in lifelong protective health and wellbeing benefits to the unborn child. However, developmental insults from sub-optimal nutrition, obesity, stress and exposure to toxins or chemicals including smoking and alcohol can increase the risk of poorer health and wellbeing [3, 4].

The effects of multi-generational health and wellbeing are also increasingly acknowledged. Although there are fewer human research studies, there is some population-based evidence, such as during the Dutch famine in the 1940s and the 1959–1961 Chinese Famine. Women who were pregnant during these famines produced smaller babies who were more susceptible in later life to diabetes and schizophrenia [5–9]. Through these studies, it has been demonstrated that the effects of diet, stress or deprivation of needs in one generation can influence the traits and characteristics of their children and even their grandchildren [10–12].

There has been a recent increase in the number of cross-generational studies; however, there are only a few that were commenced as prospective longitudinal birth cohorts (e.g. The Lifeways Cross-Generation Cohort Study [13]). This multi-generational, longitudinal approach allows for the exploration of inter-generational differences and supports a focus on the contributions of genetics, environment and lifestyle, on general health and wellbeing and other specific traits [14]. Pregnancy is a time of excitement, anticipation, adjustment, stress and anxiety for parents [15]. Then, when the baby is born, the roles and relationships between different family members also change and transition [16, 17]. Therefore, studying families across generations as a new family member enters is an ideal time to examine health, relationships and wellbeing.

The main prospective Illawarra Born cross-generation study is a multi-generational study of family health and wellbeing, in particular examining mental health and preventable chronic disease. For this main study, it is proposed to recruit three generations—the preborn baby, the parents and grandparents—and to collect numerous data from this entire cohort unit across time. This is an innovative design and provides the opportunity to simultaneously study nested cross-sectional variables within a genetic-variant family and compare between and across generations within the same study period and at longitudinal time points. Longitudinal cohort studies of individuals provide the best tool to obtain high-quality evidence for the determinants of health, by allowing the relationships between events, characteristics and subsequent outcomes to be identified and evaluated [14]. Most cohort studies follow one group of individuals (sometimes two) [18]; however, this project will be one of very few studies that follow an extended family unit over time.

The Illawarra-Shoalhaven area has many features that may be ideal for a multi-generational study. The region covers a large geographic area and has a mix of rural and metropolitan influences. Further, it has a relatively stable, cohesive population that is representative of the broader New South Wales (NSW) and Australian population [19]. However, there remain a number of health issues of particular relevance for the Illawarra community [20]. The major risk factors in the Illawarra associated with preventable mortality are tobacco use, alcohol use, physical inactivity, poor nutrition, overweight and obesity and unhealthy environments [20]. In particular, Illawarra residents are more likely than the average NSW resident (11% compared with 9.8%) to be experiencing psychological distress [21]. The study offers the opportunity to investigate and address many of these prevalent health issues across the lifespan.

Illawarra Born is a multi-disciplinary collaborative study involving researchers from the University of Wollongong (UOW) and the Illawarra Shoalhaven Local Health District (ISHLD). The research team consists of psychologists, doctors, obstetricians, paediatricians, epidemiologists, biologists, dieticians, geneticists and social scientists. The framework of the cross-generational birth cohort study allows a diverse mix of researchers to come together to explore three key research themes of importance to the community and specific researcher interests. The first research area identifies how cross-generational relationship patterns influence the mental health variables of relationship attachment, reflective functioning, mindfulness and parenting capacity. The second area explores the knowledge, attitudes and behaviours of mothers with respect to health behaviours, including folate and iodine in pregnancy. The final area examines the exposures to environmental toxins in utero and as adults. For the main Illawarra Born study, the intention is to recruit 1000 families. This pilot study was designed to test feasibility and acceptability, with the aim of recruiting a small group of pregnant women and subsequently her baby, partner and mother to test the research protocol. In addition to the previously outlined three key research areas of the main study, several sub-studies were incorporated into this feasibility study that focused on cardiovascular development, predictors of pre-eclampsia and the role of oxytocin in maternal peri-natal complications.

This Illawarra Born feasibility pilot study was the first step towards the main longitudinal birth cohort study. The feasibility study aimed firstly to generate findings on the research questions and determine sample size estimates required. Secondly, the current study was designed to evaluate the feasibility and acceptability of a longitudinal study with potential participants and stakeholders and to trial recruitment and data collection processes. In the main study, recruitment will not be time limited, but continue until the target of 1000 pregnant women have been recruited. The main study of Illawarra Born could potentially include 1000 family units and have up to 7000 participants. The main study will be over sampled to allow for multiple births and pregnancy losses. Golding [22] argues that good sample size estimates can be obtained with birth cohort study sizes between 600 and 10,000 to obtain meaningful results.

## Methods

### Aim and design

This feasibility pilot study was designed as a multi-generational birth cohort study using a multi-disciplinary-based biopsychosocial framework. The strength of this approach is that it offers a multilevel approach to understanding health outcomes, by examining individual-level psychological and biological factors, the relationship level factors, sociocultural level factors and community level factors when approaching a research inquiry [23].

### Objectives of the feasibility study

The purpose of this paper is to report on the feasibility and acceptability of the research protocol for a cross-generation health study in the community. The objectives were to determine:

- The feasibility of the research protocol; was it possible to recruit participants and collect data as planned
- The acceptability of the research protocol; was the data collection procedures and time demands suitable from the perspective of the participants
- Generate findings on the research questions
- Identify barriers to successful study completion

### Ethics

The study was approved by the Institutional Review Board and local health district prior to commencement (HE2013/377).

## Feasibility of the research protocol

Recruitment took place at the Wollongong Hospital, a level 5 unit (approved to care for women of ≥ 32 weeks gestation) that sees an average of 2500 births per year. The local health service offers a variety of models of care that include midwifery-led care through a Midwifery Group Practice (one-on-one care), midwifery-led antenatal care in outreach clinics, a general practitioner shared care model and the traditional hospital-based care [24]. There are specialty services for Aboriginal clients, adolescents, women experiencing illicit substance usage, women with special needs and those with high-risk pregnancies [24].

All pregnant women sufficiently fluent in English to be able to complete the surveys were eligible for recruitment, regardless of parity. No interpreter services were available to participants. The only exclusion criterion for recruitment was if the woman planned to move away from the Illawarra area before the birth.

In the feasibility study, midwives at the Wollongong Hospital antenatal clinic were enlisted to recruit pregnant women. Prior to the commencement of recruitment, members of the research team conducted information gathering and education sessions with midwives. Midwives then provided information about the study to pregnant women at their history-taking interview, which is a routine appointment at that particular hospital. A member of the research team was regularly present in the antenatal clinic waiting room to answer any questions that potential participants or midwives may have had. Women were asked at that visit or subsequent visits whether they were interested in finding out more information regarding the study and if they gave consent, and their phone number was passed onto the research team for follow-up. These potential participants were then sent a detailed participant information pack electronically or by mail and followed up via phone 7–10 days later.

### Data collection

Full description of the study instruments and timeline of administration is given in Tables 1 and 2. A broad range of physical and mental health data were collected from all participants at multiple time points. Where possible, standardised measures with established reliability and validity were selected as data collection tools [25, 26]. Recognised data collection tools including the NSW Population Health Survey, NSW Midwives Data Collection and Australian National Infant Feeding Survey were incorporated. This ensured the findings of the study could be examined in light of the broader NSW and Australian population health outcomes. For other research questions, new tools were developed, including diet and weaning surveys, and novel methods were included, such as accelerometers being fitted on mothers and infants for up to 7 days to measure sleep and activity. Data collection methods included physical assessments, questionnaires (online and paper-based), medical records review, observation, interviews and biological sampling. Anthropometric measures, including height and weight, and blood pressure measurements were collected at every wave. For the infants, skin fold measures at the bicep, tricep, suprailiac and subscapular locations were also collected by a trained health professional. An Illawarra Born BioBank was established for the storage of biological samples to provide for future investigations, upon participants’ consent.

**Table 1:** Pilot study biological samples

Sample type	Purpose	Maternal	Paternal	Infant	Grandmother
Blood	Erythrocyte folate, haemoglobin, Vitamin B12, serum folate, inflammation marker (C-reactive protein), iron (serum ferritin and serum transferring receptor), serum 25-hydroxyvitamin D, blood pesticide, zinc and lead levels studies	Time 2	Baseline	Birth	Baseline
	Oxytocin and cortisol levels	Time 2, 3			
	Pregnancy zone protein	Time 2, 3			
	Banking for biorepository	Time 2, 3	Baseline	Birth	Baseline
Urine	Mercury, cadmium and arsenic study	Time 2	Baseline		
	Iodine study	Time 2, 4	Baseline	Time 4	
	Congo dot test	Time 2, 3			
	Metabolite thiosulphate			Time 4	
	Banking for biorepository	Time 2, 3	Baseline	Time 4	Baseline
Faecal	Banking for biorepository			Time 3	
Cheek swab	DNA			Time 3	
Exhaled gas	Respiratory H_2_S			Time 4	
Saliva	Oxytocin and cortisol levels			Time 3

**Table 2:** Pilot study standardised assessment measures

Focus area	Instrument	Maternal completion	Paternal completion	Grandmother completion	Infant completion	Administered
Mental health						
	Depression Anxiety Stress Scale (DASS) [41]	Time 2, 3 and 4^#^				Self
	Edinburgh Postnatal Depression Scale [42]	Time 3 and 4				Self
	Kessler Psychological Distress Scale (K-10) [43]	Baseline	Baseline	Baseline		Self
Personality						
	Depressive Experiences Questionnaire (DEQ-SC6) [44]	Time 2				Self
	Difficulties in Emotional Regulation Scale [45]	Time 2, 3 and 4		Baseline		Self
	Five Facets Mindfulness Questionnaire [46]	Time 2, 3 and 4		Baseline		Self
Attachment, Bonding and Relationships						
	Adult Attachment Interview (AAI) [47]	Time 2		Baseline		Researcher
	Dyadic Adjustment Scale* [48]	Baseline, time 3, 4	Baseline	Baseline		Self
	Maternal Foetal Attachment Scale [49]	Time 2				Self
	Maternal Postnatal Attachment Scale [50]	Time 3 and 4				Self
	Measure of Parental Style (MOPS) [51]	Time 2	Baseline	Baseline		Self
	NCAST Parent Child interaction (PCI) Scales [52]	Time 3 and 4				Researcher
	Paternal Foetal Attachment Scale [53]		Baseline			Self
	Relationship Questionnaire-Clinical Version [54]	Time 2	Baseline	Baseline		Self
Quality of life						
	Prenatal Psychological Profile: Stress Scale [55]	Time 2				Self
	World Health Organisation Quality of Life (WHOQOL)-BREF [56]	Baseline	Baseline	Baseline		Self
	World Health Survey—disability [57]	Baseline	Baseline	Baseline		Self
Alcohol consumption						
	Alcohol Use Disorders Identification Test [58]	Baseline	Baseline	Baseline		Self
Childbirth and parenting						
	Infant Behavior Questionnaire—short form [59]	Time 4				Self
	Obstetric events [60]	Time 3				Self
	Parental Reflective Functioning Questionnaire [61]	Time 4				Self
	Parenting Self-Regulation Scale [62]	Time 4				Self
	Perceived control in childbirth scale [63]	Time 3				Self
	Satisfaction in childbirth scale [63]	Time 3				Self
Diet						
	24-h recall (multiple pass method)	Time 4			Time 4	Researcher
Motor development						
	Alberta Infant Motor Scale (AIMS) [64]				Time 4	Researcher

*Selected question/s

^#^Baseline for women was 22 weeks gestation, time 2 30 weeks gestation, time 3 7–10 weeks post-partum and time 4 6 months post-partum. Baseline for fathers and grandmothers was first visit

## Acceptability of the research protocol

### Data collection settings, timeline and procedures

Participants gave written informed consent following Institutional Review Board approval. Data were collected at Illawarra Health and Medical Research Institute (IHMRI), an independent health and medical research institute located on the University of Wollongong campus. IHMRI is a joint initiative of the UOW and ISLHD. The first wave of data collection began when women at 22 weeks gestation attended IHMRI at baseline. The final data collections of mothers and babies at the 6-month-old paediatric checks were also performed at the research site. There were two data collection points while participants were still pregnant, which included a survey at 22 weeks gestation (at IHMRI) (time 1—baseline) and a survey, interview and blood and urine collection for biological samples analyses at 30 weeks gestation (at IHMRI) (time 2). Where women consented, their baby’s cord blood was collected at birth by hospital staff. Following discharge from hospital, their obstetric records were obtained with consent. All of the participants agreed to the collection of biological samples from themselves and their infants once born.

At the 30 weeks gestation data collection point (time 2), women were asked if they would be willing to pass on an information pack to their partner and their mother (referred to as maternal grandmother), if relevant. Women were provided with personalised information packs to pass on, and partners and maternal grandmothers were asked to contact the research team if they would like to participate. Maternal grandmothers were selected as representative of the third generation for convenience since their pregnant daughters were the initial consenting participants in the study. This aspect of the study requested fathers and grandmothers to participate in a survey and participate in the collection of blood and urine samples. There were no exclusion criteria based on genetic relationships.

After giving birth, women were invited to participate in the post-partum stage of the feasibility study, which included two visits to IHMRI with their infant at 7–10 weeks (time 3) and 6 months post-partum (time 4). At each of these visits, a health check with a paediatrician, an interview and assessments took place. Blood samples were also collected at time 3. Following the final time 4 visit, women were given the opportunity to provide anonymous feedback to the research team through an exit survey.

### Sample size section and rationale

A recent systematic review of longitudinal birth cohort studies in Australia and New Zealand reported a median of 1095 mothers across 23 studies [14]. Therefore, for this feasibility study, a target sample of 55 women (5%) was deemed suitable.

### Planned analysis and evaluation

Participant recruitment and retention in the study across the four data collection phases was analysed by the methods outlined by Walters et al. [27], in that recruitment rate is determined by the total number of participants divided by the months. After the final data collection, women were invited to participate in an anonymous online exit interview questionnaire. Questions were asked in relation to their experiences of being involved in the study, including the degree their rights were upheld, their satisfaction with the study, demands placed upon them and their infant and feedback on the benefits of participating. The acceptability of the data procedures and time demands was collected through an anonymous survey and reported cross-sectionally at the end of the study. Research outcomes were initially examined cross-sectionally for each generation and then explored within participants longitudinally. At the feasibility stage, descriptive statistics were used to describe the three generational cohorts: infant, parent and grandparent. Missing data were managed as follows. In the case where data were missing on only one variable within a measure for a participant, Little’s Missing Completely at Random (MCAR) test [28] was conducted. The variables determined to be missing at random were then replaced within subscales using expectation maximisation. In the instance where more than two variables were missing on a scale, the data were excluded from the analysis. Barriers to successful study completion were identified by discussions during meetings with the author group, as well as the broader Illawarra Born Collaborative study group.

## Results

### Evaluating feasibility: recruitment and data collection

**Fig. 1:**
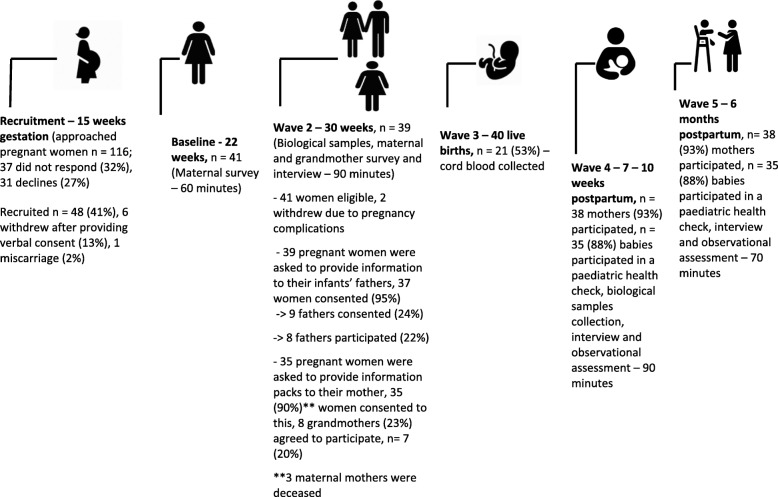
Flow of Illawarra Born maternal and infant participants

Recruitment for the study took place over an 8-month period, from April 2014 to December 2014, and the follow-ups in this pilot occurred in 2015. Figure 1 describes the flow of Illawarra Born participation. We recruited 48 women for the pilot (41% of those who were approached for participation); 41 women went on to participate in data collection. The recruitment rate was six participants per month, which was lower than expected. At time 4, 6 months post-partum, maternal retention rates were 93%. The recruitment of extended family was challenging for the research team, as shown in Fig. 1.

Table 3 describes the cohort profile of Illawarra Born mothers at recruitment and delivery and of Illawarra Born babies at the two postnatal visits. Sampling bias became apparent because recruiting disadvantaged and marginalised groups early in pregnancy was difficult, and it was the more educated women who were more likely to volunteer. The findings from the pilot sample demonstrate a variety of health and education outcomes relevant to lifespan developmental research.

**Table 3:** Selected characteristics of Illawarra Born mothers and children

Variable (continuous)	*N*	Mean (SD)	Variable (categorical)	*N*	%
Pregnancy					
Maternal age (years)	41	31.3 (4.6)	Country of birth Australia	41	88
Time to fall pregnant (months)	41	5.3 (7.5)	Previous parity	41	61
Aware pregnant (weeks)	41	4.9 (1.7)			
Pre-pregnancy BMI (kg/m^2^)	40	24.6 (4.2)	Planned pregnancy	41	78
Pregnancy weight gain (kg) hospital records	39	5.0 (1.7)	Maternal education	41	
2nd trimester systolic BP (mmHg)	39	115.3 (10.5)	Early school leaver		2
2nd trimester bloods			Completed schooling		5
Iron (μmol/L)	39	14.9 (7.6)	Trade qualification		12
Transferrin (g/L)	39	3.8 (0.06)	University or college		81
Transferrin saturation (%)	39	20.1 (11)	Household income	40	
Ferritin (μg/L)	39	16.5 (11.6)	< AUD $80,000 pa		23
C-reactive protein (CRP) (mg/L)	39	4.5 (2.9)	> AUD $80,000 pa		77
Haemoglobin (g/L)	39	118.7 (7.6)	Married	41	73
Vitamin B12 (pmol/L)	39	232.5 (60.5)	Maternal smoking history	41	
25(OH)vitamin D (nmol/l)	39	67.1 (2.9)	Non-smoker		56
Lead (μmol/L)	39	0.03 (0.02)	Smoked previously		44
Serum folate (nmol/L)	39	39.1 (8.5)	Unsafe alcohol consumption		44
Soluble transferrin receptor (mg/L)	38	1.3 (.3)	Adequate physical activity < *	41	49
2nd trimester urine	39				
Iodine (μg/L)		143.8 (13.2)			
Birth					
Birthweight (g)	40	3498.9 (409.8)	Gestational weight gain (at T2)^#^	39	
Birth length (cm)	39	50.7 (2.1)	Inadequate		59
Head circumference (cm)	40	34.7 (1.3)	Adequate		23
Gestational age (weeks)	40	39.8 (1.2)	Excessive		18
Birthweight for gestational age *z*-score			Induced labour	40	38
Cord blood	21		Caesarean delivery	40	13
Iron (μmol/L)	21	29.3 (6.9)	Instrumental birth	40	23
Transferrin (g/L)	21	2 (0.3)	Episiotomy	39	23
Transferrin saturation (%)	21	74.8 (19.7)	Post-partum haemorrhage	39	8
Ferritin (μg/L)	21	326.6 (260.9)	Perineal trauma	40	
CRP (mg/L)	21	0.4 (1.5)	Intact		25
Haemoglobin (g/L)	19	138.2 (27.2)	1st degree tear		33
25(OH)vitamin D (nmol/l)	21	87.2 (24.5)	2nd degree tear		20
Lead (μmol/L)	20	0.2 (0.01)	3rd degree tear		3
Serum folate (nmol/L)	20	44.4 (1.9)	Pre-term birth		0
Soluble transferrin receptor (mg/L)	20	2 (0.6)	Child female		48
Infancy					
BMI (kg/m^2^) at 6 months	27	16.7 (1.9)	Maternal weight retention > 5 kg at 6 months post-partum	33	12.1
Systolic BP (mmHg) at 7–10 weeks	23	91.3 (15.2)	Breastfeeding at 6 months	29	72.4
Systolic BP(mmHg) at 6 months	25	95.6 (15.0)	Introduction of solids at < 4 months	31	22.6
AIMS score	35	27.4 (9.4)	Introduction of solids at < 6 months	31	96.8

^#^30 weeks gestation

*Institute of Medicine (US) and National Research Council (US) Committee to Reexamine IOM Pregnancy Weight Guidelines. Weight Gain During Pregnancy: Reexamining the Guidelines. Washington DC: National Academy of Sciences; 2009

In a longitudinal study, a variety of sensitive information may be obtained. Key sensitive information that arose in the feasibility study related to mental health concerns. The way in which sensitive information would be dealt with was outlined in consent forms [29, 30], which stated, “In the rare instance that the research discovers a potentially serious concern, this will be managed on a case by case basis”. Examples included “the need to make a mandatory report in the case of child welfare concerns, seeking emergency mental health care if suicidal, or discussing with you and subsequently your health professional if problems were discovered in a health assessment”. A number of referrals were made during the study in consultation with the scientific committee and the participant. In collaboration with the Wollongong Hospital, clinically significant findings from biological tests obtained during pregnancy were shared with women’s health professionals with the participants’ knowledge. No findings from any genetic studies including oxytocin receptor analyses were provided in the feasibility study.

### Evaluating the acceptability of the research protocol

Twenty-nine of the women responded to the exit interview, of this group, 100% rated the quality of their experience as a participant as excellent or above average, 100% were satisfied or very satisfied with their experience as a participant in the study and all would consider participating in future research conducted by the study research team. All responding participants agreed their rights and their infant’s rights as research participants were adhered to. In relation to the ongoing requirements as a participant, one third of the group felt these were moderately demanding (*n* = 8) or unreasonably demanding (*n* = 1), while the remaining 20 women felt it was not at all demanding.

### Generating findings on the research questions

For a modest feasibility study, the research team has been able to generate several publications in the following areas: iodine supplementation use during pregnancy and lactation [31] and the influence of mindfulness and attachment styles on mother and infant interaction [32, 33]. Several further articles are currently under peer review, or the research team is in the process of responding to reviewers’ feedback. This study has also provided data for four higher degree research programs.

### Barriers to successful study completion

The first challenge was the 18 months required to obtain ethical and research site approval. This was longer than expected but potentially not uncommon [34], particularly considering the various levels of ethical approval required for cohort studies collecting biological samples [35]. Other ethical considerations were related to promoting the wellbeing of study participants and their families. This is ultimately the responsibility of the scientific committee, but it is also the responsibility of all personnel involved in the study.

The major resources to conduct the study were the salary of the research co-ordinator followed by blood processing costs, followed by participant costs. There was considerable in-kind support of two researchers who undertook sample processing and storage. The two paediatricians and the phlebotomist involved in the study provided their efforts in kind. The study cost in total $122,979 to conduct over a 22-month period, at a cost of $1281 per participant. A significant time commitment for the research team related to paper medical files. Electronic medical records were being implemented by the hospital as the study progressed; however, they did not cover all aspects of women’s care; therefore, researchers were required to access scanned paper files. The process of reviewing these files to complete obstetric records data collection form was labour intensive and took an average of 3h per participant.

## Discussion

The decision to undertake the Illawarra Born cross-generational study was worthwhile, and this pilot study demonstrated proof of concept, but some changes were required to the research protocol to support recruitment rates and reduce demands on participants as well as on the research partners. The challenges and lessons learned may assist in future work and for other researchers planning such studies.

### Feasibility of the research protocol

Recruitment of pregnant women was a significant challenge with only 41% of women who were approached actually consenting to participate. Additionally, the burden to participate was quite high with four separate visits being required by the mothers in the study, together with several biological sample collections. The research site (IHMRI) now has a facility at the Wollongong Hospital which will be used for all pregnancy and early post-partum data collection, so the mothers can align their antenatal clinic visits with participation in the study. The pathways for fathers and grandparents to enter the study will also be changed to commence with an online survey instead of a face to face visit to address identified issues related to working and commuting as well as living away from the local area.

Seeking involvement of clinicians in recruitment was a challenge, particularly when they feel overstretched within a busy birthing unit. This was despite the research team having a number of clinicians who were based at the hospital. The approach taken by the Growing up in New Zealand study where researchers had direct contact with potential participants in a range of health and community settings, in addition to the antenatal clinic, will be used in the main study [36]. A public awareness campaign will also be conducted for the main study to support a range of recruitment pathways.

### The acceptability of the research protocol

The collection and processing of biological samples and the establishment of the Illawarra Born BioBank within the health and medical research institute were successful, but there were a number of issues about the hospital-based sample collections. There was the practical issue of distance in terms of being able to meet blood processing requirements, with researchers at the university campus located 3.6 km away. The collection of cord blood was a key challenge, and despite 100% consent rates, only half of all infant cord blood was collected by health clinicians. This was despite a range of initiatives to support this collection being developed. Furthermore, the need to process the cord blood as close as possible to birth inevitably led to the researcher team being on call and having to complete work after-hours. A change in protocol was required in relation to the post-partum maternal blood collection. This was initially proposed to occur within 24 h of giving birth, but a number of samples were missed due to a significant number of women being discharged within this time in line with the hospital policy. Consequently, this post-birth data point was changed to occur at the women’s first post-partum visit at IHMRI, which is also more relevant to the time of onset for post-natal depression [37]. Being able to access electronic medical records will support the main study [38]. However, it is important to continue to collect self-report data from women as the medical records information is not always completely accurate [39].

### Generating research findings

We have demonstrated the feasibility of an institute-health partnership in studying the perinatal period as a fruitful tool to launch longitudinal research questions. The use of a multidisciplinary approach and broad biopsychosocial data collection method provides optimism for contributing to further scientific studies [14].

### Barriers to successful study completion

Partnering with clinicians in health services is crucial in undertaking research of this kind. We underestimated the time required to obtain hospital approval to undertake the study, engage with management and then clinicians and build trust before commencing the study. We also faced a number of challenges with regard to ensuring complete and timely collection of biological samples.

## Conclusion

The study established feasibility and acceptability for a planned larger longitudinal study with recommendations for methodology. Pilot families have been followed from recruitment at 15 weeks gestation to 6 months post-partum. Despite challenges outlined, the feasibility study has demonstrated the acceptability of a cross-generation health study in the community and enabled optimisation of recruitment, data collection time points and outcome measures. The commitment to undertaking a study of this kind is substantial in terms of costs and time [40], but the value is likewise.

## Abbreviations

IHMRI: Illawarra Health and Medical Research Institute
ISHLD: Illawarra Shoalhaven Local Health District
NSW: New South Wales
UOW: University of Wollongong
